# Identification of lncRNA-mRNA Regulatory Module to Explore the Pathogenesis and Prognosis of Melanoma

**DOI:** 10.3389/fcell.2020.615671

**Published:** 2020-12-17

**Authors:** Jiaqi Zhang, Hui Liu, Wenhao Zhang, Yinfang Li, Zhigang Fan, Hua Jiang, Judong Luo

**Affiliations:** ^1^Department of Radiotherapy, The Affiliated Changzhou No. 2 People's Hospital of Nanjing Medical University, Changzhou, China; ^2^Department of Dermatology, Graduate School of Dalian Medical University, Dalian, China; ^3^Aliyun School of Big Data, Changzhou University, Changzhou, China; ^4^Department of Oncology, Affiliated 3201 Hospital of Xi'an Jiaotong University, Hanzhong, China; ^5^Department of Oncology, The Affiliated Changzhou No.2 People's Hospital of Nanjing Medical University, Changzhou, China

**Keywords:** skin cutaneous melanoma, long non-coding RNA, differential expression, gene regulation, messenger RNA (mRNA), survival analysis, prognostic biomarker

## Abstract

Skin cutaneous melanoma (SKCM) is an aggressive form of skin cancer that results in high mortality rate worldwide. It is vital to discover effective prognostic biomarkers and therapeutic targets for the treatment of melanoma. Long non-coding RNA (lncRNA) has been verified to play an essential role in the regulation of gene expression in diseases and tumors. Therefore, it is significant to explore the function of lncRNAs in the development and progression of SKCM. In this paper, a set of differentially expressed lncRNAs (DElncRNAs) and mRNAs (DEmRNAs) were first screened out using 471 cutaneous melanoma samples and 813 normal skin samples. Gene Ontology and KEGG pathway enrichment analysis were performed to obtain the significant function annotations and pathways of DEmRNAs. We also ran survival analysis on both DElncRNAs and DEmRNAs to identify prognostic-related lncRNAs and mRNAs. Next, a set of hub genes derived from protein-protein interaction (PPI) network analysis and lncRNA target genes screened from starbase-ENCORI database were integrated to construct a lncRNA-mRNA regulatory module, which includes 6 lncRNAs 4 target mRNAs. We further checked the capacity of these lncRNA and mRNA in the diagnosis of melanoma, and found that single lncRNA can effectively distinguish tumor and normal tissue. Moreover, we ran CMap analysis to select a list of small molecule drugs for SKCM, such as EGFR inhibitor AG-490, growth factor receptor inhibitor GW-441756 and apoptosis stimulant betulinic-acid, which have shown therapeutic effect in the treatment of melanoma.

## 1. Introduction

Malignant melanoma is one of the most aggressive malignancies that have a strong tendency to metastasize during the early stage of the disease (Kremenovic et al., 2020). Although surgical removal of the primary melanoma can significantly improve the survival rate of patients, there are approximate 10% cases diagnosed at advanced stage, by then the tumor has become metastatic and unresectable and thus yields to poor prognosis (Domingues et al., 2018; Kremenovic et al., 2020). Some therapeutic treatments, such as targeted therapy and immunotherapy, have been verified to somewhat improve the prognosis of patients with metastatic melanoma (Bai et al., 2019). However, most melanoma patients still suffer from poor prognosis with only 5-year relative survival rate. Therefore, it is urgent to uncover effective biomarkers for early diagnosis and novel therapeutic targets of melanoma.

The initiation and progression of melanoma arise due to the separate or combined action of genetic and epigenetic factors (Fischer et al., 2018). With the development of high-throughput sequencing technique, there has been significant advance in exploring the underlying molecular mechanisms, as well as the identification of crucial signal transductions and pathways in SKCM (Ko and Fisher, 2011). Many protein-coding genes have been shown to correlate with melanoma. Several growth factors, including basic fibroblast growth factor (bFGF), epidermal growth factor (EGF), platelet-derived growth factor (PDGF), transforming growth factors (TGF), and insulin-like growth factors 1 and 2 (IGF-1 and IGF-2), have been reported to be significantly up-regulated in melanoma cells (Polsky and Cordon-Cardo, 2003; Menezes et al., 2018). Mutations in the CDKN2A gene are the most common alteration in hereditary melanoma (Aoude et al., 2015). This gene encodes the p16 protein, which inhibits cyclin-dependent kinase (CDK) 4 and 6, and reduces cell cycle arrest and apoptosis (Soura et al., 2016). Moreover, it is reported that SOX10 is as equally specific as S100 for the detection of melanoma metastases in sentinel lymph node (Szumera-Ciekiewicz et al., 2020). Besides, some well-known melanoma markers HMB-45 and Melan-A are often used in the clinical diagnosis.

Meanwhile, more and more studies have shown that long non-coding RNAs (lncRNAs) play an important role in various biological processes (Chen et al., 2019). LncRNA is a type of RNA transcripts with non-coding potentials more than 200 nucleotides, which exhibits histone modification functions similar to protein-coding genes. LncRNAs comprise the majority of transcripts in the mammalian genomes, however, their functions still remain largely unknown (Ramilowski et al., 2020). Huang et al. reported that lncRNAs may participate in every step of gene expression via transcriptional, histone modification, post-transcriptional, and/or translational regulation (Huang et al., 2018). Some lncRNAs take effect in gene regulation and thus influence various facets of cellular homeostasis, including proliferation, survival, migration, and genomic stability (Ramilowski et al., 2020). Besides, they can modulate gene expression through transcriptional activation/repression and RNA editing/splicing/degradation (Moran et al., 2012).

Therefore, an increasing number of studies have poured attention to the correlation between lncRNA and cancer. For example, it has been reported that lncRNAs play crucial functional roles in tumorigenesis, including melanoma (Huarte, 2015). In an early study that identifies the lncRNA signature of melanoma metastasis, several metastasis-associated lncRNAs were tested for their possible function in lymphatic metastasis of melanoma. As a result, relative to matched primary melanomas, lncRNA HOTAIR was found to be highly expressed in lymph node metastases (Tang et al., 2013). Moreover, it has been shown that MALAT1 is intensively related to lymph node metastasis, and UCA1 is correlated with advanced melanoma (Tian et al., 2014), which indicates that lncRNAs may be effective biomarkers for identifying metastasis of SKCM. As lncRNA functions through regulating mRNA processing and post-transcriptional regulation (Geisler and Coller, 2013), lncRNA and mRNA may influence each other, thereby constitute complex regulation layers. The process of transcription and/or splicing of the lncRNAs confers a gene-regulation functionality. Transcription of lncRNA may influence local chromatin states and transcription factor (TF) binding on promoter and enhancer regions (Kopp and Mendell, 2018). For example, it is reported that impaired transcription or splicing of Blustr changed the chromatin state of the Sfmbt2 promoter, as well as decreasing RNA polymerase occupancy at the transcription start site and within the gene body of Sfmbt2. Transcription and splicing of the Blustr RNA is closely related to the expression of neighboring gene (Engreitz et al., 2016). However, the interactions between lncRNAs and mRNAs still remain unknown in melanoma to date. Investigation of the underlying molecular mechanisms of lncRNA participating in melanoma is helpful to preventing neoplasm metastasis and improve prognosis of melanoma patients.

In this paper, we set about to investigate the interactions between aberrantly expressed lncRNA and mRNA to uncover their regulation functions in SKCM. We collected genome-wide expression profiles from TCGA and GTEx database, and conducted bioinformatics analysis to explore the function of differentially expressed lncRNAs and mRNAs. A set of key lncRNAs and protein-coding genes were identified via survival analysis. GO functional annotations and KEGG pathways were subsequently identified via gene set enrichment analysis. By integrating lncRNA targets and hub genes of the PPI network, we established an essential lncRNA-mRNA regulatory module that underlie the occurrence and progression of SKCM, to provide new insight into the mechanism of lncRNAs and potential treatment of SKCM. Moreover, connectivity map (CMap) analysis yielded to several drugs that can reverse the expression profiles of the set of key genes and thus indicate potential treatment of melanoma.

## 2. Materials and Methods

### 2.1. Data Source

To evaluate the difference of mRNA and lncRNA expressions between the diseased and normal samples, we obtained the fragments per kilobase million (FPKM) values of mRNA and lncRNA expressions from UCSC Xena (Goldman et al., 2020), covering 471 SKCM samples and 1 normal sample from The Cancer Genome Atlas (TCGA) (Tomczak et al., 2015). Besides, we obtained 812 normal samples from Genotype Tissue Expression (GTEx) (Lonsdale et al., 2013).

As a result, a total of 471 tumor samples and 813 normal samples were collected. Clinical data with complete survival information of 463 SKCM patients were also retrieved from UCSC Xena. The expression profiles included 13,840 mRNAs and 2,249 lncRNAs, and the expression levels were calibrated and standardized.

### 2.2. Differential Expression Analysis

The Limma package (version 3.44.3) (Ritchie et al., 2015) was used to identify the differentially expressed lncRNA and mRNA. The log_2_ fold change >2 and *p*-value < 0.01 were used as the criteria to screen differentially express genes. The R packages ggplot2 and pheatmap were used to plot the volcano maps and heat maps of the two groups of DEGs.

### 2.3. GO and KEGG Pathway Enrichment Analysis

The Gene Ontology (GO) and Kyoto Encyclopedia of Genes and Genomes (KEGG) analysis were conducted using the set of DEmRNAs for functional annotations and pathway enrichment analysis. The clusterProfiler (version 3.16.0) (Yu et al., 2012) was used to perform functional annotations and pathway analysis. Three type functional annotations, including biological process (BP), cellular composition (CC), and molecular function (MF), were covered. The threshold *p*-value < 0.05 was applied to select statistically significant functional terms and pathways.

### 2.4. Survival Analysis

To screen out key factors from the set of differentially expressed lncRNAs and mRNAs, survival analysis was conducted based on the melanoma cohort in TCGA. According to median levels, patients were divided into two groups with high and low expression levels. Overall survival (OS) rate analysis was then performed using the R package survival (Therneau and Lumley, 2014), based on which the OS differences between the two groups were estimated. Finally, the Kaplan Meier method (Efron, 1988) with a log-rank test was applied to evaluate the significance of difference, and the genes with *p*-value < 0.01 were considered statistically significant.

### 2.5. PPI Network and Hub Gene Identification

The protein-protein interactions (PPIs) were derived from STRING database (Szklarczyk et al., 2016). Cytoscape (Smoot et al., 2011) greatly facilitates the PPI analysis by readily visualizing the PPI network, in which each node represents a protein or a gene and an edge represent the interactions between two nodes. The degree of a node is the number of their connections with other nodes. Based on the PPI network, we used MCODE^1^ to screen out the important modules and then used cytoHubba^2^ to calculate the degree value. Only the nodes with degree score ≥20 were regarded as important ones.

### 2.6. Connectivity Map-Based Drug Screening

The connectivity map (CMap) is a large-scale online database that contains whole genomic expression profiles of cultured cancer cells treated with bioactive small molecules, including the drugs with clinical utility, known mechanism of action, or nomination from the NIH Molecular Libraries Program (Subramanian et al., 2017). By submitting the set of deregulated genes derived from PPI analysis to CMap web server, we obtained a list of drugs with connectivity scores scaled from –100 to 100, which indicate the functional connections among drugs, genes, and diseases. A positive score implies that an agent might facilitate the expression of the gene signature, while a negative score indicates that an agent might repress or reverse the expression of the gene signature (Zhang et al., 2020).

### 2.7. Construction of lncRNA-mRNA Network

Starbase-ENCORI (Li et al., 2014), an platform for studying the miRNA-ncRNA, miRNA-mRNA, and ncRNA-RNA interactions from CLIP-seq, degradome-seq and RNA-RNA interactions, was used to screen target genes of the DElncRNAs. The standard for selection of the genes was that the number of interactions is no less than 2 and at least 1 experiments had been performed. The overlapped mRNAs between the DEmRNAs and the lncRNA-targeted mRNAs were selected and used to construct the lncRNA-mRNA regulatory module. The Cytoscape was also employed to visualize the generation of the network.

## 3. Results

### 3.1. Differentially Expressed mRNAs Correlated to Melanoma

**Figure 1:**
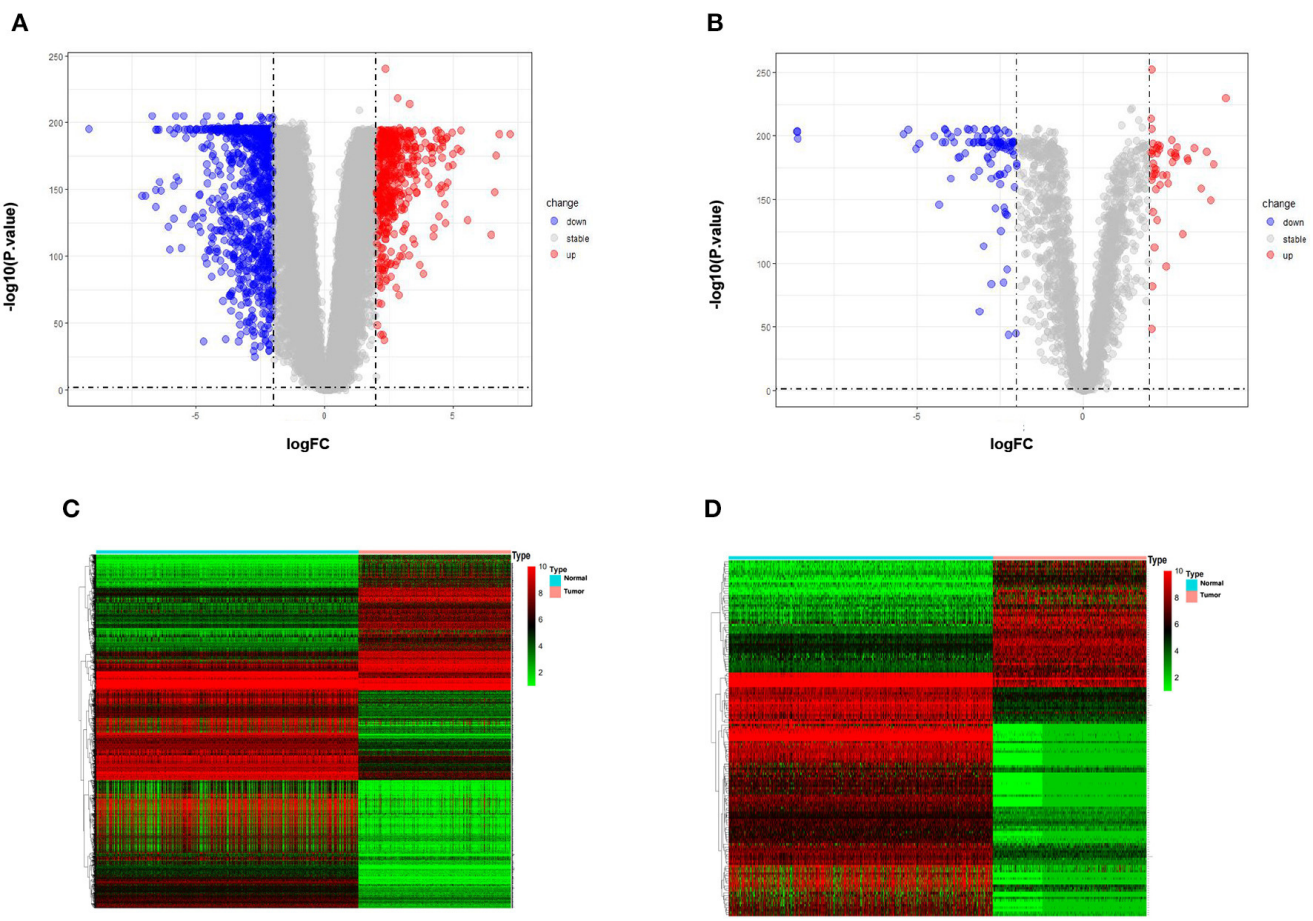
Differentially expressed genes between normal samples and melanoma patients (*log*2 fold change>2, *p*-value < 0.01). **(A)** Volcano plot of the *p*-value as a function of weighted fold change for DEmRNAs. **(B)** DElncRNAs, red dots represent significantly upregulated expressed genes and green dots represent significantly downregulated expressed genes (*log*2 fold change>2, *p*-value < 0.01). **(C)** Heat map for potential mRNAs (*n* = 1,485) showed significant expression changes, in which 971 were downregulated and 514 were upregulated. Red through green color indicates high to low expression level. **(D)** Heat map for potential lncRNAs (*n* = 146) showed significant expression changes, in which 46 were downregulated and 100 were upregulated.

The differential expression analysis revealed 146 lncRNAs and 1,485 that are differentially expressed between tumor and normal samples. Among them, 100 lncRNAs and 514 mRNAs are up-regulated in SKCM, and 46 lncRNAs and 972 mRNAs are down-regulated. As is illustrated in Figure 1, the differentially expressed lncRNA and mRNAs are visually displayed in the form of volcano maps and heat maps. The top 20 significantly deregulated lncRNAs and mRNAs are shown in Tables 1, 2, respectively.

**Table 1:** TOP 10 up-regulated/down-regulated lncRNAs in melanoma compared to normal samples.

**lncRNA**	**p-value**	**Function/phenotype annotation**
AC055720.2 ↑	7.01E-253	Diseases associated with renal cell carcinoma
BANCR ↑	1.77E-230	BRAF-activated non-protein coding RNA: plays a role in tumor progression and epithelial to mesenchymal transition
AL445250.1 ↑	2.57E-214	Alzheimer's disease, mean corpuscular hemoglobin and opioid dependence
AC096677.1 ↑	3.32E-206	Pulse pressure measurement and aortic stenosis
AC012321.1 ↓	4.42E-206	Blood osmolality measurement
AC034102.4 ↓	4.42E-206	Novel transcript, antisense to PA2G4: body mass index, Alzheimer's disease, age at onset
AC005523.2 ↓	4.42E-206	Novel transcript, antisense to FEM1A: diseases associated with melanoma
AC008738.7 ↓	4.42E-206	Heel bone mineral density
AL138478.1 ↓	1.20E-205	Acute myeloid leukemia
AC012615.3 ↓	1.94E-205	Novel transcript, antisense to KLF16: erythrocyte count, mean corpuscular and hemoglobin
AL160408.2 ↓	3.26E-205	Total cholesterol measurement and erythrocyte count
AC007620.2 ↓	3.26E-205	Response to antidepressant
AL583722.2 ↓	3.26E-205	Novel transcript, antisense to AKT1
AC006027.1 ↓	3.26E-205	Novel transcript, antisense to NOD1
AC073896.4 ↑	1.15E-194	Novel transcript, sense overlapping to SMARCC2: abnormality of refraction, macula measurement and myopia
AC007546.2 ↑	1.30E-193	Cognitive function measurement and schizophrenia
BAIAP2-DT ↑	1.78E-191	Response to allopurinol, gout, uric acid measurement, diseases associated with cervical squamous cell carcinoma and hepatocellular carcinoma
PIK3CD-AS2 ↑	5.31E-191	Irritable bowel syndrome and blood protein measurement
AL513165.1 ↑	5.80E-190	Macrophage inflammatory protein 1b measurement
MIR3142HG ↑	2.22E-188	Melatonin metabolism and effects

**Table 2:** TOP 10 up-regulated/down-regulated mRNAs in melanoma compared to normal samples.

**lncRNA**	**p-value**	**Function/phenotype annotation**
TRIM51 ↑	2.92E-241	Tripartite motif-containing 51: protein ubiquitination and innate immune response
CPN1 ↑	2.46E-219	Carboxypeptidase N polypeptide 1: peptide metabolic and bradykinin catabolic process
CSAG1 ↑	2.57E-215	Chondrosarcoma associated gene 1: encoding of a member of a family of tumor antigens and expression of chondrosarcomas
TOMM6 ↓	4.41E-206	Translocase of outer mitochondrial membrane 6: metabolism of proteins and mitophagy
XBP1 ↓	4.42E-206	X-Box binding protein 1: the regulation MHC class II genes, DNA-binding transcription factor activity and sequence-specific DNA binding
OVCA2 ↓	4.42E-206	Ovarian tumor suppressor candidate 2: hydrolase activity, diseases associated with ovarian cancer and duplication syndrome.
TREX1 ↓	4.42E-206	Three prime repair exonuclease 1: DNA repair and serve as a proofreading function for DNA polymerase
KREMEN1 ↓	4.42E-206	Kringle Containing Transmembrane Protein 1: apoptotic process and Wnt signaling pathway
C1QTNF5 ↓	4.42E-206	C1q And TNF related 5: components of basement membranes and play a role in cell adhesion
CMC4 ↓	9.67E-206	C-X9-C motif containing 4: involvement in some t(X;14) translocations associated with mature T-cell proliferations
ADIRF ↓	1.32E-205	Adipogenesis regulatory factor: regulation of lipid metabolism by peroxisome proliferator-activated receptor alpha (PPARalpha) and developmental biology
CEMP1 ↓	8.84E-205	Cementum protein 1: development of the periodontium and cementum
BOLA2 ↓	2.84E-204	BolA family member 2: acts as a cytosolic iron-sulfur (Fe-S) cluster assembly factor and be involved in iron maturation
OPN1SW ↑	8.38E-197	Opsin 1, short wave sensitive: encoding of the blue cone pigment gene
MAPRE1 ↑	9.60E-196	Microtubule associated protein RP/EB family member 1: regulation of microtubule structures and chromosome stability
CNPY3 ↑	1.26E-195	Canopy FGF signaling regulator 3: binds members of the toll-like receptor protein family, folding and export of these proteins
SDC3 ↑	3.46E-195	Syndecan 3: affecting the actin cytoskeleton, glycosaminoglycan biosynthetic and catabolic process
CCDC167 ↑	3.51E-195	Coiled-coil domain containing 167: integral component of membrane
RAP2B ↑	4.02E-195	RAP2B, member of RAS oncogene family: signal transduction and negative regulation of cell migration
TPMT ↑	4.62E-195	Thiopurine methyltransferase: the enzyme that metabolizes thiopurine drugs

**Figure 2:**
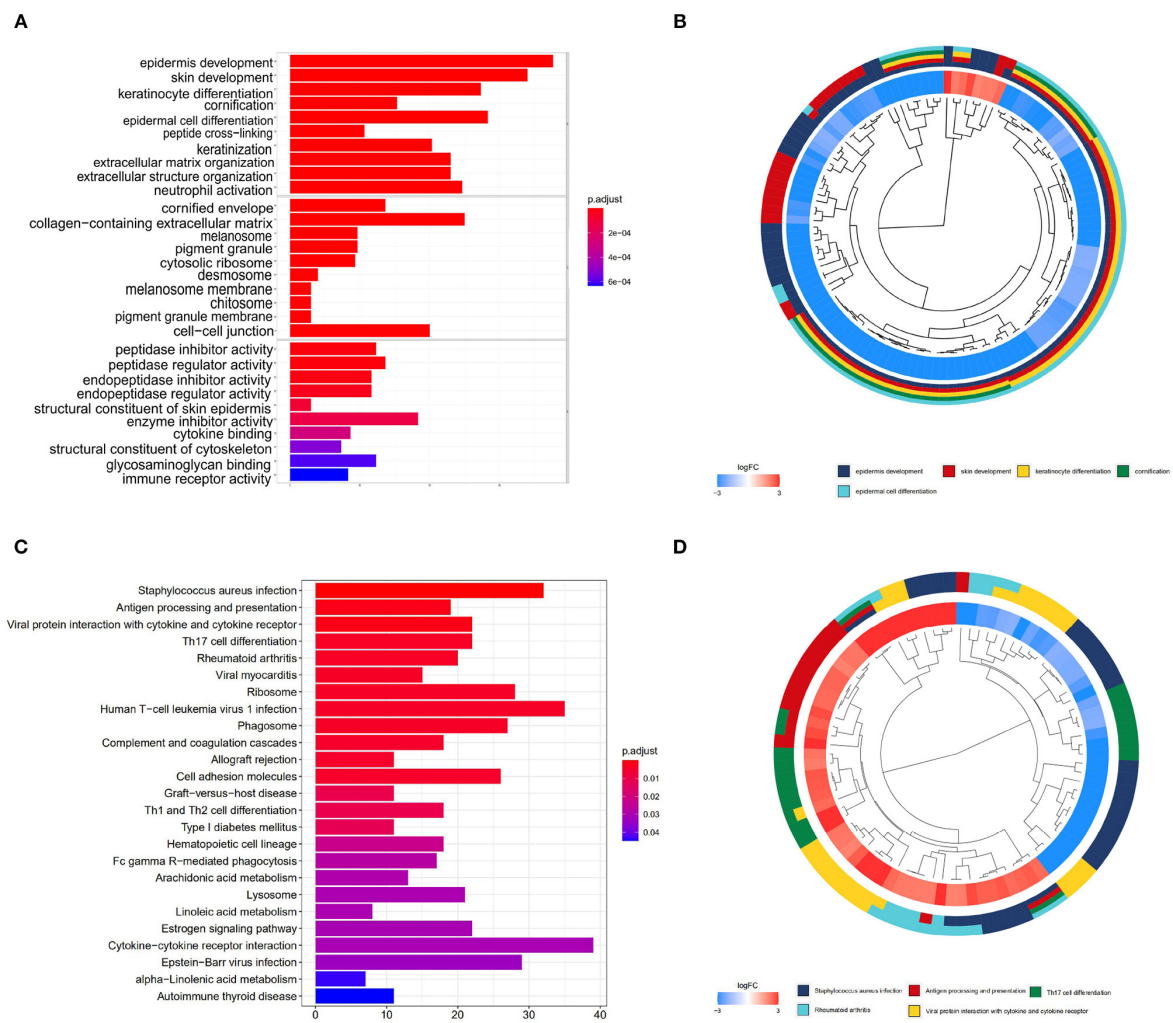
GO and KEGG enrichment analyses. **(A,B)** show the bar plot and cluster plot of significant GO funtional items. **(C,D)** show the bar plot and cluster plot of significant KEGG pathways.

To uncover the role of the differentially expressed mRNAs (DEmRNAs) in the pathogenesis of melanoma, GO enrichment analysis was carried out. As shown in Figure 2, DEmRNAs are significantly enriched in skin epidermis-related biological processes, such as epidermis and skin development, keratinocyte differentiation, cornification, and epidermal cell differentiation. As for cellular component, these DEmRNAs are significantly enriched in the cornified envelope, collagen-containing extracellular matrix, melanosome, and pigment granule. For molecular function, they are significantly enriched in peptidase and endopeptidase-related activity and structural constituent of skin epidermis, as shown in Figures 2A,B. These enrichment analysis results indicate that DEmRNAs associated with epidermal cell structure and differentiation might contribute to melanoma tumorigenesis. For example, Kodet et al. report that melanoma cells are able to influence locally the differentiation pattern of keratinocytes *in vivo* as well as *in vitro*. They have shown that FGF-2, CXCL-1, IL-8, and VEGF-A participate in the activity of melanoma cells on keratinocytes (Kodet et al., 2015). Moreover, the extracellular matrix, which was known as major component of the local microenvironment, is commonly deregulated and becomes disorganized in cancer (Lu et al., 2012).

According to the KEGG enrichment analysis, top 25 significantly enriched pathways are involved in the process of cancerogenesis. More specifically, DEmRNAs are mainly enriched in pathway related to immune and inflammatory response, including *Staphylococcus aureus* infection, antigen processing and presentation, viral protein interaction with cytokine and cytokine receptor. They are also involved in the Th17 cell differentiation and rheumatoid arthritis, as shown in Figures 2C,D. These results strongly indicate that the DEmRNAs may play important role in melanoma by involving in the inflammation and immune response.

### 3.2. Differentially Expressed lncRNAs and mRNAs Associated to Survival Rate

**Figure 3:**
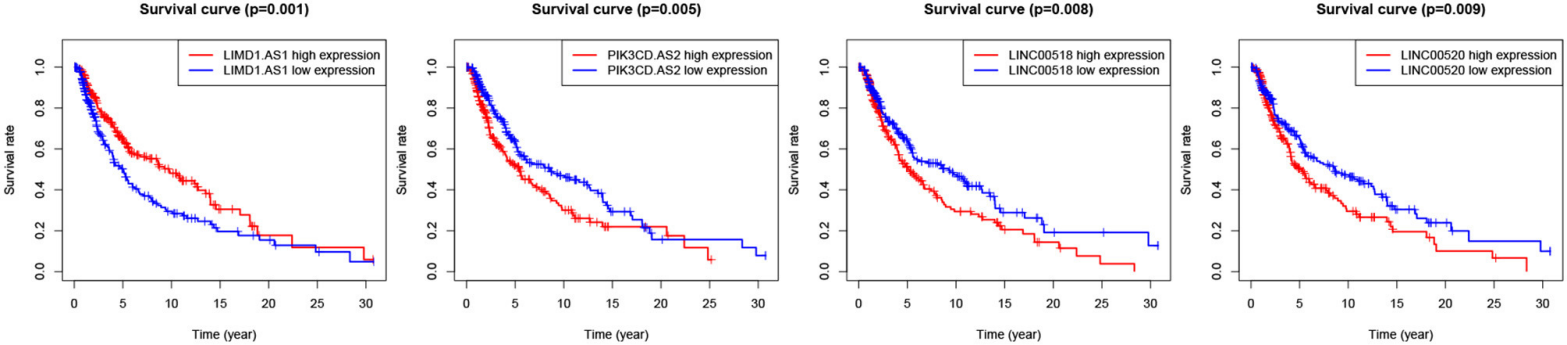
Survival curves of four lncRNAs with the significant logrank *p*-value.

We evaluated the relationship between DElncRNAs and the clinical outcome of patients with melanoma. By performing Kaplan Meier survival curves analysis based on the survival data from TCGA, We found more than 10 lncRNAs were statistically significant in association with patient survival rate. The survival curves of the most significant 4 lncRNAs are illustrated in Figure 3. LIMD1-AS1 (*p*-value = 0.00137) signature has been shown to predict prognostic survival and direct clinical risk-specific treatments in melanoma (Liu et al., 2019). LINC00518 (*p*-value = 0.00803) has shown to act as a competing endogenous RNA to promote the metastasis of malignant melanoma via miR-204-5p/AP1S2 axis (Luan et al., 2019). Also, LINC00520 (*p*-value = 0.00931) promotes the proliferation and metastasis of malignant melanoma by inducing the miR-125b-5p/EIF5A2 axis (Luan et al., 2020). Another weighted gene co-expression network analysis show that the PI3K subunit PI3KCD exhibited excellent efficacy for diagnosing primary and metastatic tumor tissue (Wang et al., 2019).

We also checked whether the DEmRNAs were statistically associated to patient survival rate. The top eight significant mRNAs are shown in Supplementary Figure 1. The expression profiles of GBP4 (*p* = 3.131e-08), FCGR2A (*p* = 5.825e-08), APOL1 (*p* = 1.408e-07), HLA-DRA (*p* = 4.035e-7), and HLA-DRB1 (*p* = 5.24e-07) are positively correlated with the survival rate, while NCCRP1 (*p* = 9.205e-08), RABIF (*p* = 3.505e-7), and FGD1 (*p* = 3.917e-07) are correlated with adverse prognosis. Among these genes, several have been reported to be associated with the prognosis of SKCM. Take GBP4 as an example, it has been verified that its high expression, together with GBP1, GBP2, GBP3, and GBP5, was correlated with favorable overall survival (OS) in the SKCM patients followed for a over 30-year study (Wang et al., 2018). Also, Brunner et al. identified GBP4 as one of the predictive genes whose expression are correlated with OS in cutaneous melanoma (Brunner et al., 2013). Also, the immune regulation pathway that FCGR2A participates in was reported to show significant association with cutaneous malignant melanoma risk (Yang et al., 2009). The functional mechanism study of FCGR2A shows that it binds more avidly to human IgG1 and IgG2 subtypes, thereby increases ADCC-mediated cell death and improve clinical outcomes (Vargas et al., 2018). The expression of class II MHC antigens, including HLA-DRA and HLA-DRB, has been chronically considered a crucial step in immune response toward colorectal carcinomas (Lee et al., 2012). Furthermore, the analysis showed that HLA-DR expression in melanoma cells may be a biomarker for tumors primed with activated T-cells and an appropriate IFNγ response to mediate sensitivity to PD-1/PD-L1 blockade, which is also associated with immune response (Johnson et al., 2016). For the genes related to adverse prognosis, NCCRP1 was also identified as an independent risk prognostic predictor for metastatic melanoma (Sun et al., 2019). FGD1 has been verified that it plays a direct role in the proliferation or invasion of melanoma cells (Hou et al., 2012), thereby the patients with high FGD1 expression frequently showed worse prognosis (Zeng et al., 2020). The complete results of the survival analysis for DElncRNAs and DEmRNAs are shown in Supplementary Table 1.

### 3.3. lncRNA mRNA Regulatory Module

**Figure 4:**
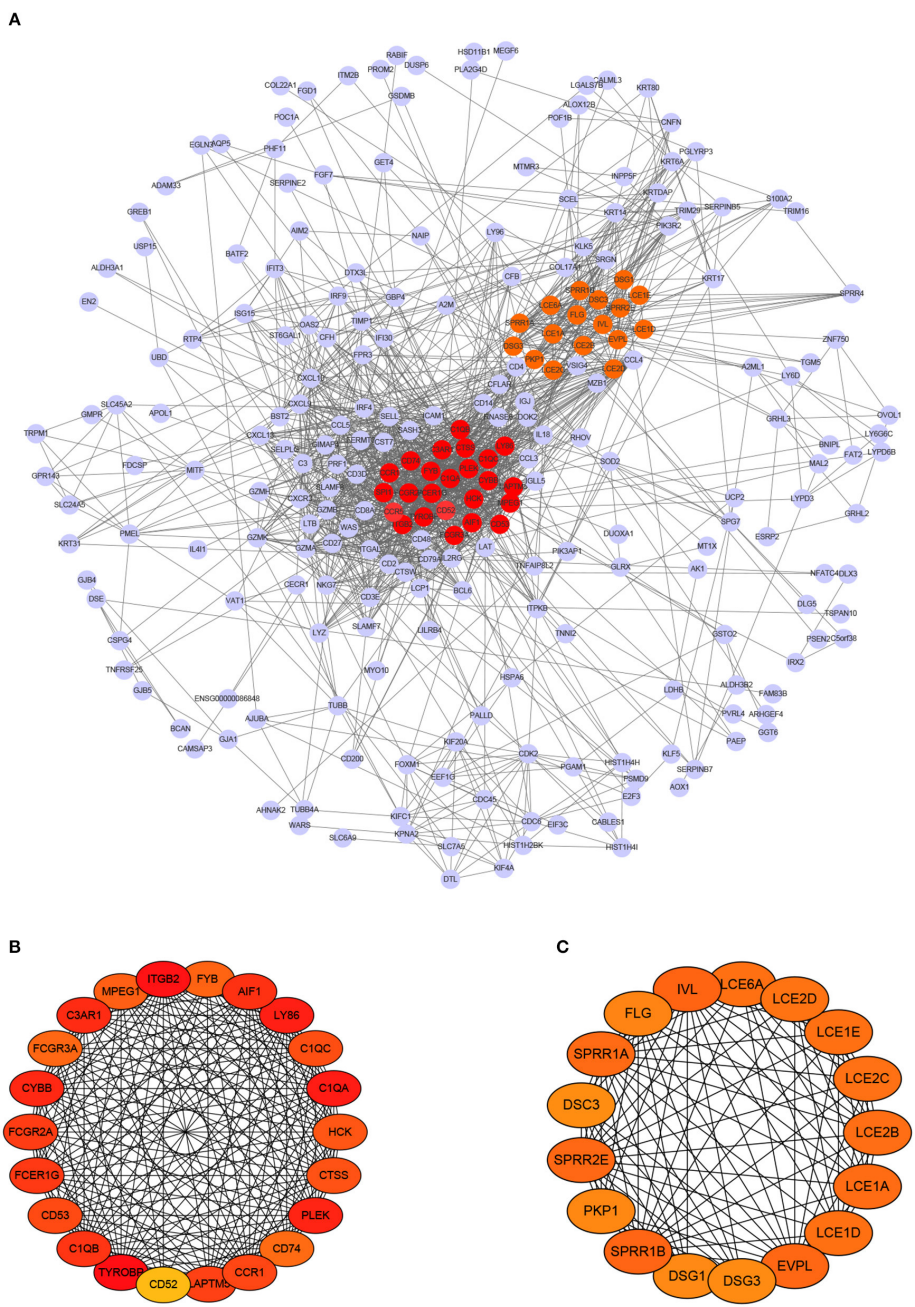
PPI network of differentially expressed mRNAs and module analysis. **(A)** PPI network contains 272 nodes and 1,577 edges. **(B,C)** display two significant modules identified by MCODE, respectively.

PPI network analysis was conducted to reveal crucial genes that are systematically related to melanoma and interconnections among them. Based on the 332 DEmRNAs that have significance in survival and the PPIs derived from STRING, 252 genes were further selected out by using Cytoscape. As a result, a PPI network that contains 252 nodes and 1,566 edges was constructed, as shown in Figure 4A.

Next, we chose hub genes that have associations with many other genes and play key role, as they are considered as potential drivers of the development of diseases (Xu et al., 2020). This step yielded to 39 hub genes, including 22 up-regulated genes and 17 down-regulated genes. Meanwhile, two modules were found out. The first module consists of 22 nodes and 215 edges (score = 20.476), and the second consists of 17 nodes and 103 edges (score = 12.875), as shown in Figures 4B,C, respectively. Note that the darker a node color, the higher its degree.

**Figure 5:**
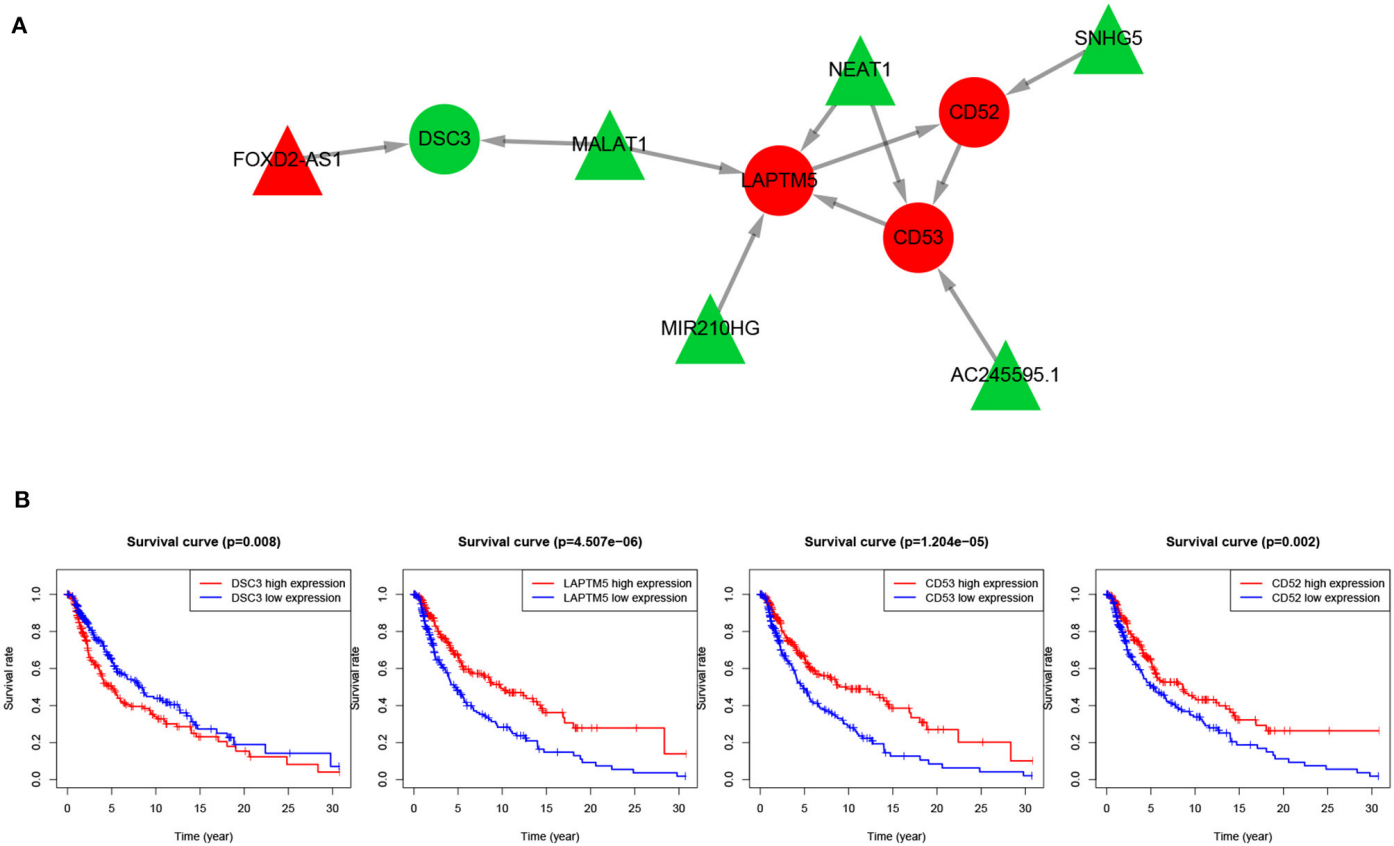
lncRNA-mRNA regulatory network and survival curves of four hub genes. **(A)** LncRNA-mRNA regulatory module, in which the red nodes represent up-regulated genes and green nodes represent down-regulated genes, triangles represent lncRNAs and circles represent mRNAs. **(B)** Survival analysis of the four overlapping mRNAs.

To further explore the key regulatory factors in SKCM, target genes of the DElncRNAs were predicted by using Starbase-ENCORI database. We got 3,069 unique genes after removal of duplicates, and found four genes (CD52, CD53, DSC3, and LAPTM5) overlapped with the set of DEmRNAs. Subsequently, we constructed a lncRNA mRNA regulatory module that consists of 10 nodes (6 lncRNAs and 4 mRNAs) and 8 edges, as is shown in Figure 5A. The survival curves of the four key genes are shown in Figure 5B. The expression levels of CD52, CD53, and LAPTM5 are positively correlated with the survival rate, while DSC3 is correlated with adverse prognosis.

We have conducted extensive literature search to verify the lncRNA mRNA regulatory module. The lncRNA NEAT1 has been reported to facilitate melanoma cell proliferation, migration, and invasion via regulating miR-495-3p and E2F3 (Xia et al., 2019). In fact, NEAT1/miR-23a-3p/KLF3 has been found to form a novel regulatory axis in melanoma cancer progression (Ding et al., 2019). FOXD2-AS1 has also been found to be upregulated in cutaneous melanoma tissue specimens and cell lines compared in normal tissue and normal human epidermal melanocyte. Inhibition of FOXD2-AS1 can suppress cutaneous melanoma cell proliferation, migration and invasion through regulating phospho-Akt expression (Ren et al., 2019). Furthermore, MALAT1 downregulates miR-34a expression level in melanoma cells and tumor specimens (Li et al., 2019), thereby it is a potential biomarker for many human cancer diagnosis as well as prognosis (Amodio et al., 2018; Li et al., 2018).

In this regulatory module we found that the expression of most key lncRNAs and their target mRNAs are negatively regulated. First, lncRNA is often located in the upstream promoter region of coding gene and inhibit the expression of adjacent genes. Second, Trans-acting lncRNAs may also function by modulating the activity or abundance of proteins or RNAs to which they directly bind. Third, lncRNA exists on the antisense strand of the coding gene, which can interfere with the splicing of mRNA, thereby produce different forms of isoforms. lncRNA may also produce endogenous siRNA under the action of dicer enzyme to regulate gene expression level. In addition to RNA-binding proteins, lncRNAs also have the ability to regulate the abundance or activity of microRNA (miRNA), a category of transcripts termed competing endogenous RNAs (ceRNAs) (Kopp and Mendell, 2018).

Although lncRNAs mainly inhibit the expression level of their target mRNAs, our results demonstrated that not all the key lncRNAs and their target mRNAs are negatively correlated. For example, in our lncRNA-mRNA regulatory module, lncRNA MALAT1 can regulate the downstream gene LAPTM5 that is actually up-regulated, meanwhile MALAT1 may also down-regulated gene DSC3. In addition, it has been shown that the down-regulation function of MALAT1 restrain the development of uveal melanoma via inhibition of HOXC4. Therefore, we suppose that the regulatory relationship between lncRNA and mRNA is not absolutely positive or negative.

Also, previous studies have shown that some of the deregulated mRNAs are involved in melanoma. For example, DSC3 was upregulated in primary melanoma than in metastatic melanoma, its high expression level was correlated with adverse prognosis. and thus considered as new biomarkers in the therapeutics of metastatic melanoma (Sheng et al., 2020). Salerno et al. reported that DSC3 is associated with the lack of Th1 immune signatures in human melanoma metastases (Salerno et al., 2016). CD53 has significant homology to melanoma-specific antigen ME491 sequence (Bellacosa et al., 1991). Also, the expression of LAPTM5 closely related to tumorigenesis in many human cancers (Nuylan et al., 2016). For example, Somura et al. report that a 3-gene predictor which contains LAPTM5 may predict the early intrahepatic recurrence of hepatocellular carcinoma (Somura et al., 2008).

### 3.4. Key lncRNAs Function as Biomarker of Melanoma

**Figure 6:**
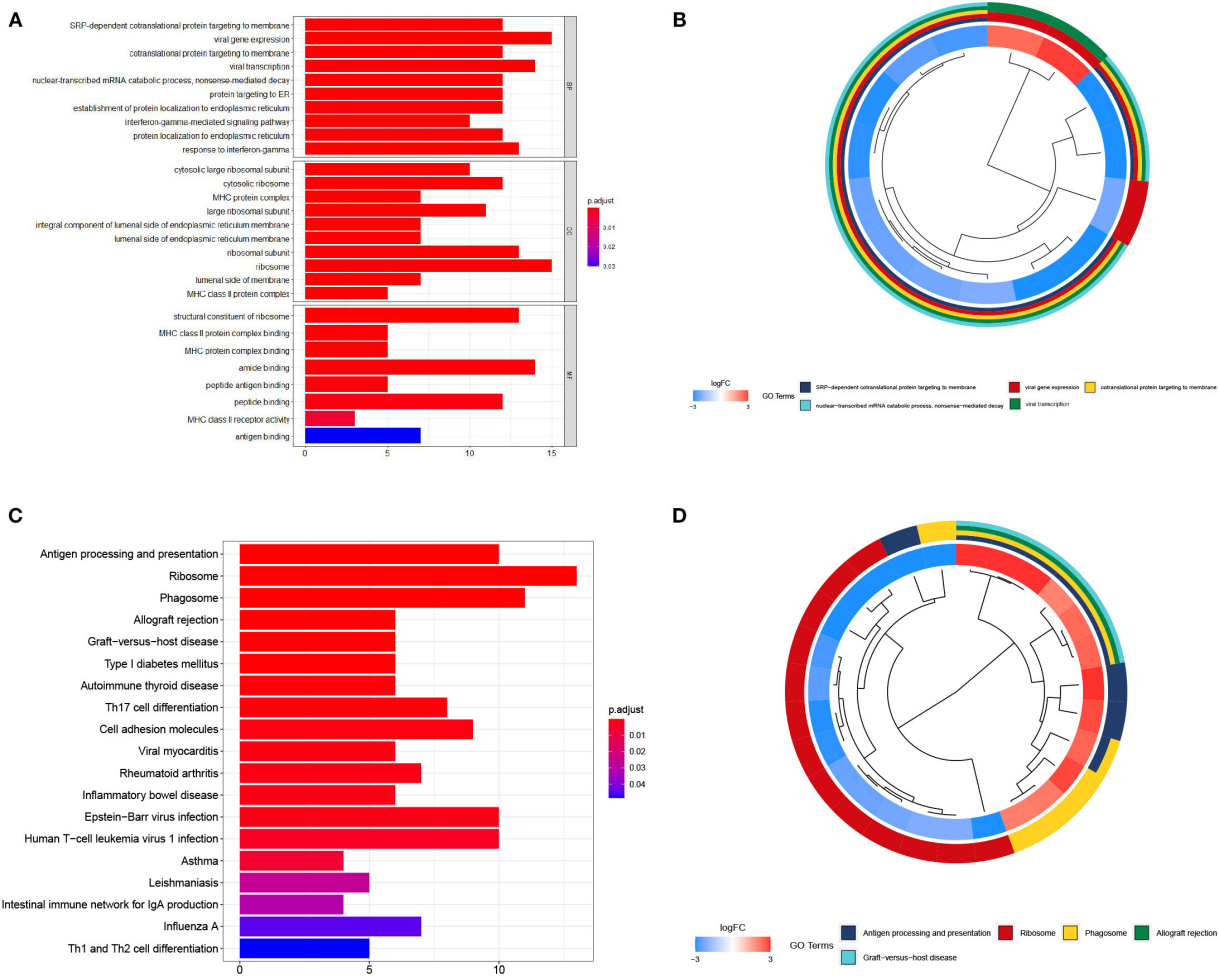
GO and KEGG enrichment analyses of key lncRNAs targeting mRNAs. **(A,B)** show the bar plot and cluster plot of significant GO funtional items. **(C,D)** show the bar plot and cluster plot of significant KEGG pathways.

To reveal the role of the key lncRNAs in the pathogenesis of melanoma, enrichment analysis was carried out by target genes of them. In terms of GO enrichment analysis results, the set of target genes are participated in biological processes such as SRP-dependent cotranslational protein targeting to membrane and viral gene expression. For cellular component, these target genes are significantly enriched in the cytosolic ribosome, large ribosomal subunit and MHC protein complex. As for molecular function, they are significantly enriched in structural constituent of ribosome and MHC class II protein complex binding, as shown in Figures 6A,B.

As for KEGG enrichment analysis, top 25 significantly enriched pathways are shown in Figures 6C,D. LncRNA target genes are significantly involved in the process of antigen processing and presentation, ribosome, phagosome and allograft rejection pathway. The enrichment analysis results powerfully indicate that these genes may contribute to melanoma tumorigenesis. There is evidence for dysregulation of ribosome biogenesis in cancers. Ribosome expression is closely connected to cell growth and proliferation. The number of ribosomes per cell is proportional to the growth rate of that cell (Scull et al., 2019). MHC-II play an important part in antigen presentation to CD4+ T-lymphocytes and anti-tumor immunity, increasing evidence indicates that tumor-specific MHC-II related to favorable outcomes in patients with cancer, including those treated with immunotherapies, and with tumor rejection in murine models (Axelrod et al., 2018). These results strongly indicate that the DEmRNAs may play important role in cancerogenesis.

**Figure 7:**
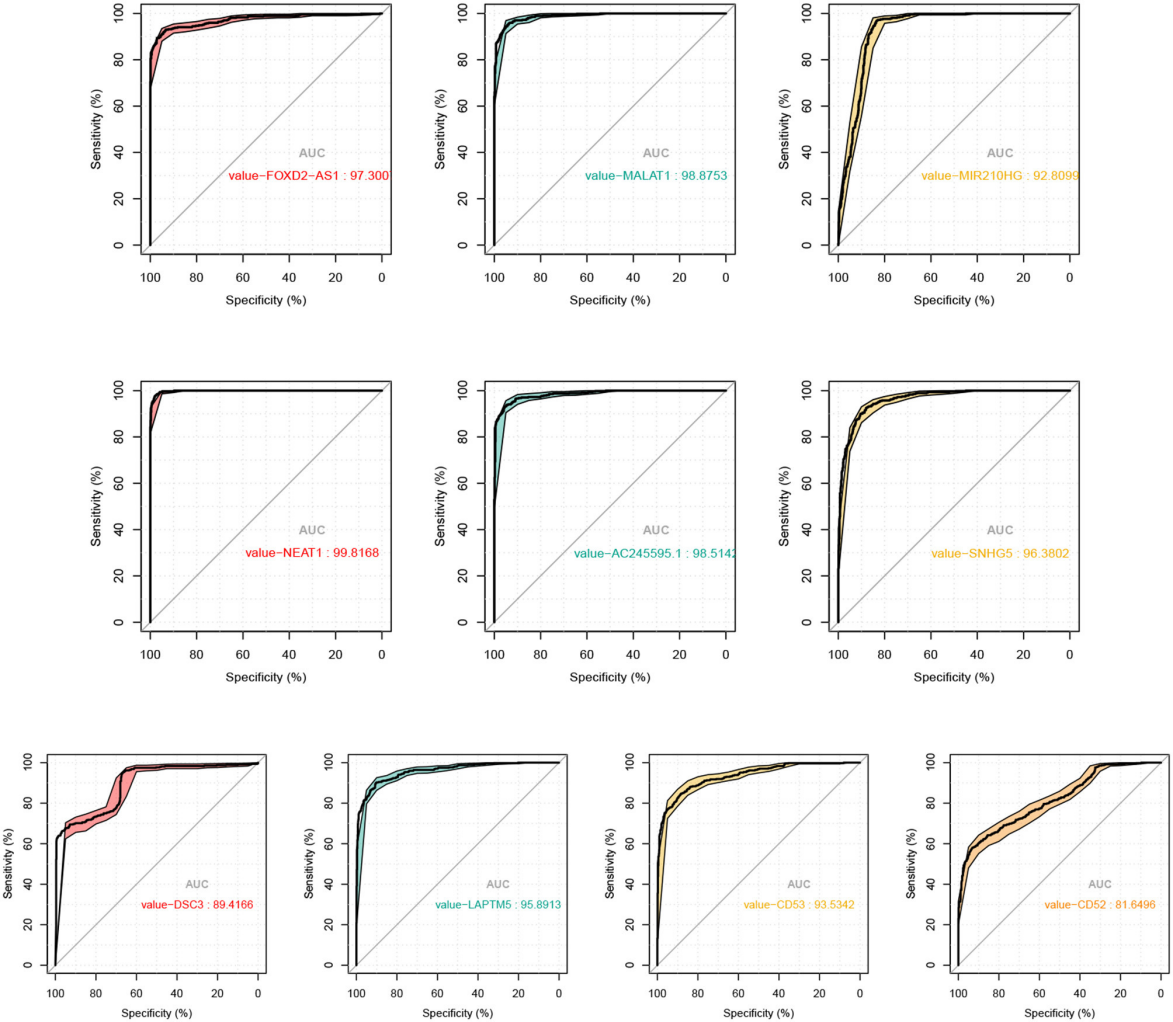
ROC curves and the corresponding AUC values of the 6 lncRNAs and 4 mRNAs as biomarker of SKCM.

To demonstrate the effectiveness of six lncRNAs as biomarker for diagnosis of melanoma, we used the expression levels of each lncRNA to distinguish tumor and normal tissues. With the varying threshold, we can build the ROC curve with respect to each lncRNA and computed the AUC values, as shown in Figure 7.

In fact, the average AUC of these regulatory factors reaches 0.97, among which FOXD2-AS1 (AUC: 0.973), MALAT1 (AUC: 0.989), NEAT1 (AUC: 0.988), AC245595.1 (AUC: 0.985), and SNHG5 (AUC: 0.964) are the most significant lncRNAs to predict diagnosis in SKCM. Previous studies have shown that these factors involved in melanoma. For example, Ren et al. have revealed that melanoma cell proliferation, migration and invasion can be suppressed by the inhibition of FOXD2-AS1 that regulates phospho-Akt expression in cutaneous melanoma. This loss-of-function study indicated that FOXD2-AS1 functions as oncogenic lncRNA in cutaneous melanoma cells (Ren et al., 2019). Also, the role of MALAT1 in metastasis of melanoma is a hot topic that raises much attention. Tian et al. have verified that knockdown of MALAT1 can attenuate the migrational ability of melanoma cells via *in vitro* studies as early as 2014, indicating the correlation between MALAT1 and melanoma metastasis (Tian et al., 2014). Thereafter, Luan et al. reported that MALAT1 promotes malignant melanoma growth and metastasis by sponging miR-22 (Luan et al., 2016). Sun et al. subsequently showed that MALAT1 can promote the proliferation, invasion and migration of melanoma cells by suppressing miR-140 expression (Sun et al., 2016). Another more recent study revealed that MALAT1 promotes melanoma development by downregulating miR-23a (Wang et al., 2020). NEAT1 was also proved to facilitate the melanoma cell proliferation, migration, and invasion via regulating miR-495-3p and E2F3 (Xia et al., 2019). SNHG5 plays an analogous role in melanoma by regulating the miR-26a-5p/TRPC3 pathway (Gao et al., 2019). In summary, all these findings strongly support these key lncRNAs are closely related to melanoma, and can be used as biomarker for diagnosis of melanoma.

### 3.5. CMap Analysis Reveals Potential Drugs

We went further to explore the small molecule drugs that can reverse the expression levels of 39 identified hub genes so that the development and progression of melanoma can be potentially inhibited. We submitted the set of genes to the Connectivity Map web server and choose skin melanoma cell line A375 to screen drugs. The returned drugs were prioritized according to their scores, and negative score indicates that drugs might repress or reverse the expression of the key genes. The top 15 small molecules with negative scores are listed in Table 3.

**Table 3:** Predicted small molecule drugs by CMAP analysis.

**ID**	**Name**	**Score**	**Description**
BRD-K24201553	SB-269970	–99.98	Serotonin receptor antagonist
BRD-A45664787	Iloprost	–99.91	Platelet aggregation inhibitor
BRD-K53570330	Carbofuran	–99.87	Cholinesterase inhibitor
BRD-K16551401	PNU-22394	–99.81	Serotonin receptor agonist
BRD-K12357156	AG-490	–99.80	EGFR inhibitor
BRD-K74430258	1,2-Dichlorobenzene	–99.76	Hepatotoxicant
BRD-A00993607	Alprenolol	–99.75	Adrenergic receptor antagonist
BRD-K12120659	GR-144053	–99.74	Integrin antagonist
BRD-K04146668	GW-441756	–99.72	Growth factor receptor inhibitor
BRD-K45401373	Betulinic-acid	–99.53	Apoptosis stimulant
BRD-K32977963	Eugenol	–99.42	Androgen receptor antagonist
BRD-K07888107	Depudecin	–99.41	HDAC inhibitor
BRD-K11163873	Phenanthridone	–99.34	PARP inhibitor
BRD-K98684188	GSK-0660	–98.97	PPAR receptor antagonist
BRD-A48720949	Testosterone	–98.86	Androgen receptor agonist

Expectedly, quite a few small molecules have been approved to inhibit the development of SKCM. For instance, EGFR inhibitor AG-490 can effectively suppress tumor cell proliferation by limiting the expression of cyclin D1 (Kamran et al., 2013). Betulinic acid has been demonstrated to induce programmed cell death with melanoma cells. Betulinic acid treatment of cultured UISO-Mel-1 (human melanoma cells) leads to the activation of p38 and stress activated protein kinase/c-Jun NH(2)-terminal kinase which widely accepted as proapoptotic mitogen-activated protein kinases (MAPKs) (Tan et al., 2003). In addition, HDAC and PARP inhibitors are also well-known as tumor-targeted drugs. More importantly, the potential effectiveness of these molecules implies that the set of hub mRNAs, based on which the CMAP analysis was conducted, are highly relates to the incidence and progression of SKCM, indicating the rationality of the bioinformatics pipeline presented in this paper.

## 4. Conclusion and Discussion

In this paper, we aim at exploring the underlying regulation network regarding to lncRNA and mRNA in SKCM using bioinformatics analysis. Firstly, a total of 146 lncRNAs and 1,485 mRNAs that are differentially expressed in SKCM tissues are identified. We use the set of DEmRNAs to perform Gene Ontology and KEGG enrichment analysis for exploring relevant biological functions at transcriptional level. The functional and pathway annotations demonstrate that the aberrantly expressed genes participate in melanoma-related biological processes, such as epidermis and skin development, keratinocyte differentiation, and cornification. Also, cellular compositions and molecular functions are related to cornified envelope, collagen-containing extracellular matrix, melanosome and peptidase inhibitor activity (Gajewski, 2007).

Next, survival analysis reveals a number of DEmRNAs that exhibit significant effects on melanoma patient survival rate, and PPI network analysis yields to 39 hub genes and two significant modules are identified. Finally, a central lncRNA mRNA module that may underlie the complex regulatory relationship between lncRNAs and mRNAs in melanoma is constructed. The lncRNA mRNA regulatory module including six key lncRNAs and four key genes are presented. We have found that the lncRNAs can be used as biomarker that effectively distinguish the tumor and normal tissue. Also, the connectivity map analysis screens a few small molecule drugs that can reverse the expression profiles of the 39 hub genes. Furthermore, our extensive literature mining also verifies that these lncRNAs and mRNAs essentially participate in the pathogenesis and prognosis of melanoma. These results demonstrate the effectiveness of our constructed lncRNA-mRNA regulatory network. We believe this regulatory module would provide novel insights of the biological mechanism in SKCM.

A limitation of our study mainly lies in that most of the data used in the bioinformatic analysis are obtained from public datasets, without verification of *in vitro* or *in vivo* biochemical experiments. Despite this limitation, we are the first to identify differentially expressed genes in skin melanoma by integrating the data of TCGA and GTEx databases. With the constructed lncRNA mRNA regulatory module, we can further comprehensively characterize the lncRNAs and mRNAs associated with melanoma, and provide new insight into the regulatory mechanism for melanoma.

## Data Availability Statement

The original contributions presented in the study are included in the article/Supplementary Materials, further inquiries can be directed to the corresponding author/s.

## Author Contributions

JZ and HL conceived the main idea and analyzed the data. JZ and WZ drafted the manuscript. HL reviewed drafts of the paper and improved the manuscript. YL collected the data and performed the statistical analysis. ZF provided statistical advice. HJ and JL supervised the study and provided funding. All authors read and commented on the manuscript.

## Conflict of Interest

The authors declare that the research was conducted in the absence of any commercial or financial relationships that could be construed as a potential conflict of interest.

## Footnotes

^1^http://apps.cytoscape.org/apps/MCODE

^2^http://apps.cytoscape.org/apps/cytoHubba
