# Supplementary figures and images for: Identification of lncRNA-mRNA Regulatory Module to Explore the Pathogenesis and Prognosis of Melanoma
Source: Front Cell Dev Biol. 2020 Dec 17;8:615671. doi: 10.3389/fcell.2020.615671 (PMC7773644; doi:10.3389/fcell.2020.615671)

# Graphs

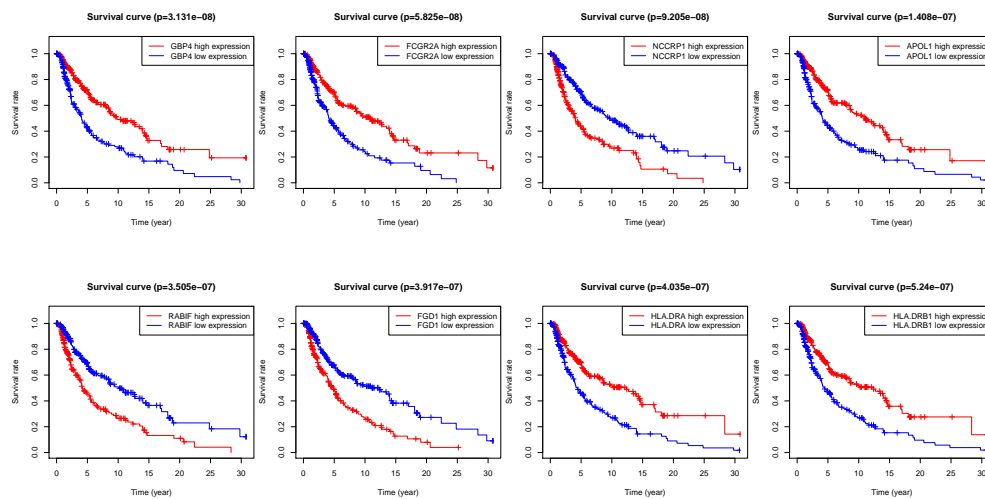

Figure S1: Survival curves of the top eight genes with most significant p-value.

Supplement: Supplementary file 1 [file Data_Sheet_1.PDF]
